# Selection in Australian Thoroughbred horses acts on a locus associated with early two-year old speed

**DOI:** 10.1371/journal.pone.0227212

**Published:** 2020-02-12

**Authors:** Haige Han, Beatrice A. McGivney, Gabriella Farries, Lisa M. Katz, David E. MacHugh, Imtiaz A. S. Randhawa, Emmeline W. Hill

**Affiliations:** 1 Plusvital Ltd, The Highline, Dun Laoghaire Business Park, Dublin, Ireland; 2 UCD School of Agriculture and Food Science, University College Dublin, Dublin, Ireland; 3 UCD School of Veterinary Medicine, University College Dublin, Dublin, Ireland; 4 UCD Conway Institute of Biomolecular and Biomedical Research, University College Dublin, Dublin, Ireland; 5 School of Veterinary Science, University of Queensland, Gatton, Australia; University of Iceland, ICELAND

## Abstract

Thoroughbred horse racing is a global sport with major hubs in Europe, North America, Australasia and Japan. Regional preferences for certain traits have resulted in phenotypic variation that may result from adaptation to the local racing ecosystem. Here, we test the hypothesis that genes selected for regional phenotypic variation may be identified by analysis of selection signatures in pan-genomic SNP genotype data. Comparing Australian to non-Australian Thoroughbred horses (*n* = 99), the most highly differentiated loci in a composite selection signals (CSS) analysis were on ECA6 (34.75–34.85 Mb), ECA14 (33.2–33.52 Mb and 35.52–36.94 Mb) and ECA16 (24.28–26.52 Mb) in regions containing candidate genes for exercise adaptations including cardiac function (*ARHGAP26*, *HBEGF*, *SRA1*), synapse development and locomotion (*APBB3*, *ATXN7*, *CLSTN3*), stress response (*NR3C1*) and the skeletal muscle response to exercise (*ARHGAP26*, *NDUFA2)*. In a genome-wide association study for field-measured speed in two-year-olds (*n* = 179) SNPs contained within the single association peak (33.2–35.6 Mb) overlapped with the ECA14 CSS signals and spanned a protocadherin gene cluster. Association tests using higher density SNP genotypes across the ECA14 locus identified a SNP within the *PCDHGC5* gene associated with elite racing performance (*n* = 922). These results indicate that there may be differential selection for racing performance under racing and management conditions that are specific to certain geographic racing regions. In Australia breeders have principally selected horses for favourable genetic variants at loci containing genes that modulate behaviour, locomotion and skeletal muscle physiology that together appear to be contributing to early two-year-old speed.

## Introduction

Thoroughbred horseracing is a global sport, with regional-specific population genetic differences which may result from variation in the racing ecosystem. Each region is responsible for determining the race ‘pattern’ which includes the grading of races (*i*.*e*. Group race status) and the determination of race distances. Racing varies across regions with respect to race distance distributions [1], racetrack surfaces and the timing of the racing calendar. The training of horses also varies because of the difference in climatic conditions, such that many horses are exercised at earlier, cooler hours of the day in many parts of Australia. Because of this, the genetics of certain sire lines may not be suitable for success in all racing regions.

An increasing number of studies in domestic animal populations focus on highly differentiated loci that have been subject to artificial selection. This approach can identify genes subject to selection that has occurred during domestication, breed formation or as a result of subsequent directional breeding for culturally desirable or economically important traits [2–10]. Alleles may increase in frequency in a population due to genetic drift (neutral variation) or selection, which if acting on beneficial mutations is referred to as positive selection [11]. These changes contribute to population adaptation and phenotypic diversity. As beneficial mutations increase in frequency towards fixation, there is a tendency towards reduced variation at neighbouring genomic regions enabling assessment of selection dynamics using high-density genetic markers.

In the horse, microsatellite markers have previously been used to identify genomic regions that have contributed to the gross anatomical, metabolic and physiological adaptations of the extreme athletic phenotype among Thoroughbreds [2]. Applied to genome-wide SNP genotypes, selection signature tests have been successful in detecting loci that are responsible for major phenotypic traits among horse populations [12] including the key genes associated with sprinting performance (*MSTN*) [13], gaitedness (*DMRT3*) [14] and height (*LCORL*) [3, 15]. Other studies have revealed selection signatures for reproduction traits [7] and morphological phenotypes [9, 16].

The discovery of the same selected genomic region using multiple population genetics-based approaches [17–29], provide convincing evidence for selection pressure on a locus. Following this idea, several composite selection tests have been developed to increase the power to detect selection such as Composite of Multiple Signals (CMS) [25], Meta-analysis of Selection Signals (Meta-SS) [4] and Composite Selection Signals (CSS) [6]. CSS uses fractional ranks of constituent tests allowing a combination of the evidence of historical selection from a set of selection tests [4, 6].

Compared with genome wide association studies (GWAS) that are commonly used to identify genes or genomic regions contributing to a trait of interest, selection signature tests: 1) can detect selection if the advantageous allele is already fixed, while GWAS fails in such a situation [30]; 2) may help to identify quantitative trait loci with small effect sizes incapable of being detected by GWAS; and 3) allow the fitness effects of many phenotypic variants with small selection coefficients to produce a detectable signal in patterns of DNA polymorphism at the underlying loci [31].

Here, using the CSS approach we aimed to identify genetic loci contributing to positive local adaptation to racing conditions in Australia. We tested the hypothesis that regional nuances of racing have led to phenotypic variation in Thoroughbreds across geographic regions and that regional phenotypic variation may be identified by analysis of selection signatures in pan-genomic SNP genotype data. The aim of the study was to identify the major genetic loci contributing to regional phenotypic variation in the Australian Thoroughbred population.

## Results and discussion

### Signatures of selection in the Australian Thoroughbred

To test the hypothesis that regional-specific phenotypic variation is underpinned by genes on which positive selection is acting, we compared allele frequency distribution variation among two data sets comprising Australian Thoroughbreds (*n* = 49) and non-Australian Thoroughbreds (*n* = 50), sampled in Europe, South Africa and USA. Principal component analysis (PCA) of the genetic relatedness matrix and between group identity by state (IBS) comparisons were performed to evaluate population structure. While there was overlap between the two groups on plotting PC1 *versus* PC2 (PCA), there was also some observable separation (S1 Fig). The IBS results indicated that the relatedness between the two groups was significantly lower than relatedness within the two groups (*P* = 3.0 × 10^−5^), indicating that while there is clearly genomic sharing among the populations, there is sufficient differentiation to warrant investigation of loci that may be variable between the two groups. To validate the CSS approach among relatively small sample sizes, we used distance as phenotype and identified the second highest score in the region on ECA18 flanking *MSTN*, the ‘speed gene’, for elite Thoroughbreds raced in short distances (*n* = 50) *versus* those raced in long distances (*n* = 50) (S1 Table) (S2 Fig).

**Fig 1:**
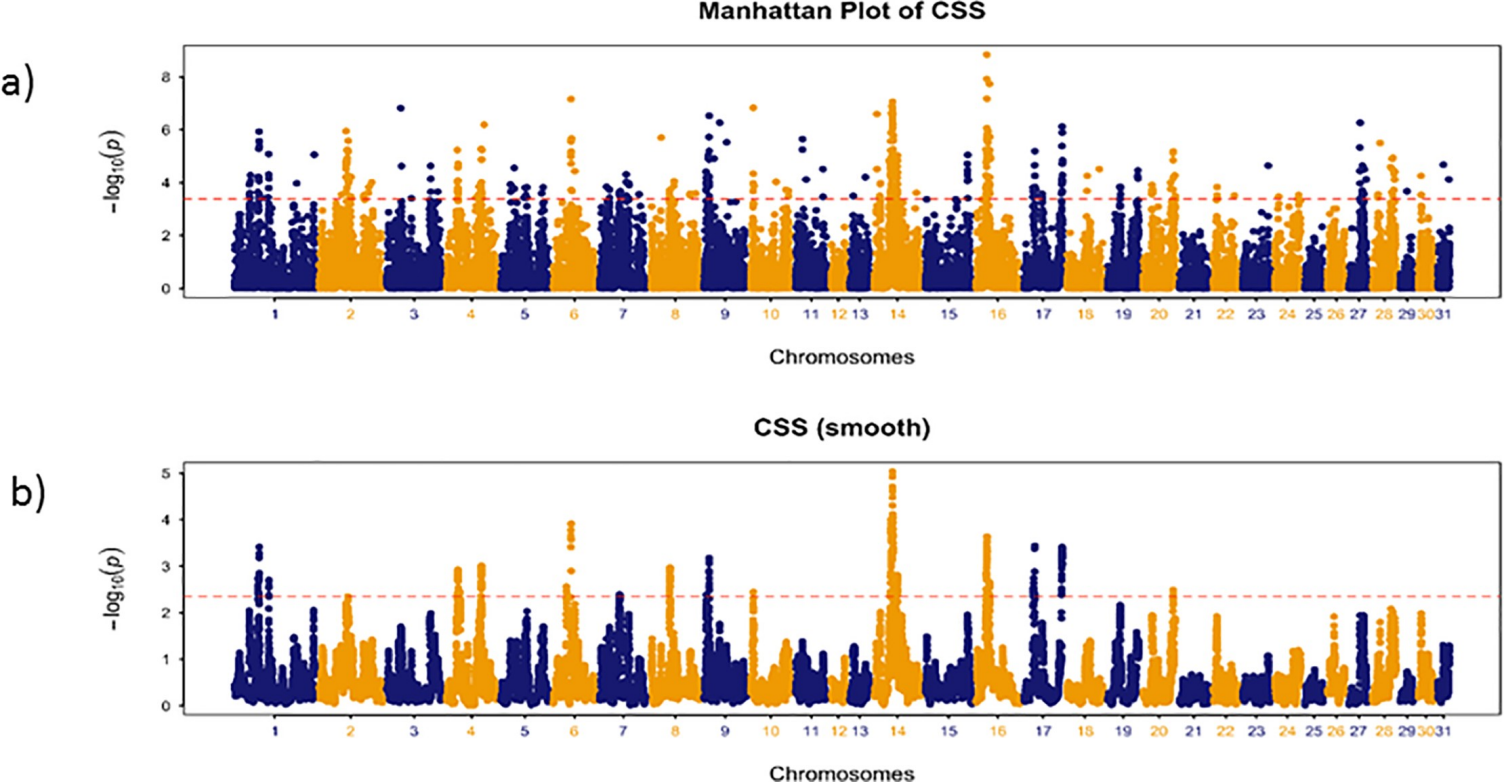
Manhattan plots of CSS and CSS (smooth) results for the Australian versus non-Australian identification of selection signals for Australian the racing phenotype. The strongest signal was on ECA14 at a 5Mb (32.17–37.8 Mb) locus containing 51 genes including multiple protocadherin genes. The highest ranked SNP was closest to the *PCDHB15* gene.

Genome-wide distribution of the smoothed CSS (-log_10_*P*) for the comparison of the Australian *versus* non-Australian populations identified three genomic regions with clusters of significant SNPs among the top 0.1% SNPs (Table 1, Fig 1, S2 Table) on ECA6 and ECA14. The top ranked region by CSS score (ECA14, 35.52–36.94 Mb) spanned ~1.5 Mb, proximal to a cluster of protocadherin genes, and contained 33 genes (S2 Table) including *APBB3* (amyloid beta precursor protein binding family B member 3 gene), *HBEGF* (heparin binding EGF like growth factor gene), *NDUFA2* (NADH:ubiquinone oxidoreductase subunit A2 gene) and *SRA1* (steroid receptor RNA activator 1 gene). The top three SNPs (14:36414548, 36308621, 36309843) flanked *SRA1* (14:36312352–36318423), which has a key role in a range of biological processes including myogenesis and steroidogenesis, and has been implicated in obesity [32]. In humans *SRA1* is associated with cardiac myopathy and in zebrafish knockdown of *SRA1* results in reduced cardiac function specifically relating to impaired cardiac contractility [33]. The second and third ranked SNPs are intronic variants within *APBB3* (14:36305566–36311548), which encodes a protein that binds the beta-amyloid precursor protein (APP), a major contributor to Alzheimer’s disease (AD). Enrichment for the KEGG pathway *hsa05010*:*Alzheimer's disease* in the skeletal muscle transcriptional response to exercise has been demonstrated in our laboratory (*P* = 2.60 × 10^−8^) [34] and the positive effects of exercise on AD are well documented [35]. Furthermore, APP is best known for its association with AD and is thought to play a role in locomotion [36], having been identified as a critical determinant of the pattern of motor neurons and neuromuscular junctions in zebrafish [36].

**Table 1:** Selected genomic regions among top 0.1% SNPs identifying core genes targeted by selection for the Australian racing phenotype.

ECA	Region (Mb)	Top 0.1% SNPs (n)	Top CSS value	Cluster rank	Cluster genes (n)	Candidate genes	Gene function
14	35.52–36.94	28	5.03	1	33	*APBB3*	locomotion
						*HBEGF*	cardiac function
						*NDUFA2*	skeletal muscle exercise response
						*SRA1*	cardiomyopathy
14	33.2–33.52	9	4.00	2	0	*NR3C1*	stress response
						*ARHGAP26*	skeletal muscle
6	34.75–34.85	9	3.91	3	1	*CLSTN3*	browning of adipose tissue; synapse development

The SNPs were ranked by the CSS score to define regions under selection since composite scores have been shown to have greater power to detect selection signals compared to any of the individual constituent tests of selection signatures [25, 37–41]. For example, in the development of the CSS statistic the three component tests (*F*_ST_, ΔSAF and XP-EHH) were found coinciding in the candidate gene regions but with fewer and lower ranked SNPs as compared to the CSS test [38]. Nonetheless, each constituent test can distinguish selection from neutrality but will be informative on the different characteristics (*i*.*e*. direction of selection and length of time) that have shaped the selection. Here, among the top 1% SNPs the highest ranked 25 SNPs by CSS score were within the ECA14 region. While three of the top CSS SNPs also had the highest XP-EHH signals, 12 of the top 20 ranking XP-EHH SNPs were in the ECA16 region (24.3–26.5Mb) that contained *ATXN7* (ataxin-7 gene) (Table 2), indicating this region has likely been influenced by recent selection. The extended haplotype homozygosity method detects genomic regions that have undergone recent selection following a rapid rise in frequency of a beneficial mutation in a relatively few generations, in a time frame in which recombination has not had time to disrupt the original haplotype.

**Table 2:** Selected genomic regions among top 1% SNPs identifying core genes targeted by selection for the Australian racing phenotype (top 3 regions only).

ECA	Region (Mb)	Top 1% SNPs (n)	Top CSS value	Cluster rank	Cluster genes (n)	Candidate genes	Gene function
14	32.17–37.48	115	5.03	1	64	*APBB3*	locomotion
						*HBEGF*	cardiac function
						*NDUFA2*	Skeletal muscle exercise response
						*SRA1*	cardiomyopathy
						*NR3C1*	stress response
						*ARHGAP26*	skeletal muscle
6	34.4–35.28	17	3.91	2	22	*CLSTN3*	browning of adipose tissue; synapse development
16	24.28–26.52	52	3.64	3	9	*ATXN7*	coordination of locomotion

The ΔSAF metric identifies highly differentiated SNPs and indicates the difference in selected allele frequency between cohorts and is particularly useful in the absence of knowledge of the ancestral allele. ΔSAF is based on the allele frequency differences between the populations and, based on the observed allele frequency distributions, the directional change in the selected allele frequency is detected [38]. The ΔSAF tends to indicate the SNP closest to a functional variant since it identifies extremes in allele frequency differences. Here the top ranked ΔSAF SNPs were in the ECA6 region closest to *PEX5* (peroxisomal biogenesis factor 5 gene), which functions in lipid metabolism in skeletal muscle [42] and *CD163L1* (CD163 molecule like 1 gene) which has been linked to creatine kinase and lactate dehydrogenase levels [43] that are commonly used as markers of muscle tissue damage [44]. On ECA14 the top ΔSAF SNPs were located within *ANKHD1* (ankyrin repeat and KH domain containing 1 gene) and *APBB3*. Since the ECA14 region defined by the CSS score is large (1.5Mb) the position of the top ΔSAF SNPs may point to the location of genes most likely to be driving selection at the locus. On ECA17 the top ΔSAF SNPs were closest to *SUCLA2* (succinate-coA ligase ADP-forming beta subunit gene), a critical component of mitochondrial pyruvate metabolism and the citric acid (TCA) cycle, and *HTR2A* (5-hydroxytryptamine receptor 2A gene), a serotonin system gene that is closely related to *HTR7* and *HTR1A*, genes that have been previously linked to precocity and tractability traits in young Thoroughbreds [45, 46]. The top ΔSAF ECA8 SNPs were closest to *CEP192* (centrosomal protein 192 gene) which is regulated by oxygen availability to control cell cycle progression in hypoxic conditions [47].

The *F*_ST_ test statistic captures the increase in highly differentiated loci among populations. Extreme positive values of *F*_ST_ for a particular locus are indicative of high levels of reproductive isolation of the two populations and divergent selection in both or strong positive selection in one of the populations and/or random drift. We did not identify ‘extreme’ (*F*_ST_ >0.5) *F*_ST_ values, the highest was on ECA14 (14:36214206, *F*_ST_ = 0.10), but this was not unexpected since the populations are not reproductively isolated. The introduction of ‘shuttle’ stallions in the 1990s that breed during both hemisphere breeding seasons, has ensured that there is continuous gene flow between the populations, but this does not preclude selection acting on phenotypic traits that are beneficial to one or another population.

Here, to identify loci under selection in the Australian Thoroughbred we focused principally on the composite CSS signal score. Given that positive selection at a specific genomic locus tends to reduce (‘sweep’) variation across a larger region, it can be difficult to identify the gene targeted by selection. Notwithstanding this, supporting evidence from complementary studies, including our previous transcriptomics analyses of the skeletal muscle response to exercise and training, may assist the identification of candidate genes driving selection at the loci identified in the current study. For example, *NDUFA2* (14:36253868–36255844), located 50 kb from the top ranked SNP, has previously been identified as an influential gene in the equine skeletal muscle transcriptome response to exercise and training as determined by network analysis of RNA-seq [34]. *NDUFA2*, which encodes a subunit of NADH:ubiquinone oxidoreductase (complex 1), had the greatest influence in the response to training network, ranking third (also *GABARAPL1* and *NDUFA6*) among all expressed genes in resting skeletal muscle. The node with the highest degree value in both the exercise and training states was *NDUFA6*, with other bottleneck genes across both the untrained and trained network states that included several genes that also encode subunits of NADH:ubiquinone oxidoreductase (complex 1) (*NDUFA4*, *NDUFA6*, *NDUFB3*, *NDUFV3*). NADH:ubiquinone oxidoreductase (complex 1) is the first large protein complex of the electron transport chain that catalyses the transfer of electrons from NADH to coenzyme Q_10_ (CoQ_10_). We have previously shown that horses with the *MSTN* g.66493737 SNP C/C genotype produce significantly more endogenous skeletal muscle CoQ_10_ than T/T horses [48], which may reflect variation in the requirement for certain substrates, or may indicate a genetically-programmed deficiency in the production of CoQ_10_ with functional consequences on exercise responses. The identification of *NDUFA2* in this study implicates this gene as a key target for selection and suggests that skeletal muscle CoQ_10_ production is a trait of importance for the Australian Thoroughbred phenotype. It has been hypothesised that increased skeletal muscle CoQ_10_ should result in more efficient skeletal muscle energy transduction [49].

Examination of expression QTL (eQTL) at the ECA14 region in skeletal muscle identified *ARHGAP26* (Rho GTPase activating protein 26 gene, GRAF-1, 14: 33978983–34396100) to be a strong candidate on which selection is acting (S4 Table). Thirty-four significant (*P* < 0.05) *cis*- and *trans*-eQTL were identified in the ECA14 region in resting, post-exercise and post-training equine skeletal muscle samples [45] (S4 Table), of which 12 were associated with expression of *ARHGAP26*; the strongest association was for TBIEC2-266584 in the untrained resting cohort (nt34490992, *P* = 6.27 × 10^−12^). GRAF-1 regulates muscle growth and maturation [50] by facilitating myoblast fusion [51] and functions in the repair of mechanically damaged skeletal and cardiac muscle cells [52].

In human skeletal muscle, exercise upregulates *HBEGF* (14: 36492030–36499880), which encodes the HB-EGF protein that acts as an insulin sensitizer and facilitates peripheral glucose disposal [53]. Overexpression of HB-EGF in a mouse model resulted in selective use of carbohydrate rather than fat as an energy substrate. The constitutive expression of HB-EGF in rat skeletal muscle suggests it has important housekeeping roles [54]. In Thoroughbred skeletal muscle, *HBEGF* was not differentially expressed following a single bout of intense exercise in untrained skeletal muscle, but rather appeared to be responsive to repetitive bouts of exercise training [34]. In Thoroughbred skeletal muscle *HBEGF* was among the most highly differentially expressed genes (98^th^ percentile) in the transcriptional response to training (1.8 fold decrease in gene expression, *P* = 7.51 × 10^−6^) [34]. In cattle, a testosterone analog stimulates the proliferation of muscle satellite cells via a response involving HB-EGF and EGFR [55]. It is unclear, in this context, why *HBEGF* would be downregulated in equine skeletal muscle following a period of training; however, in the mouse no differences in HB-EGF mRNA or protein expression were observed in skeletal muscle of rats following functional overload of muscle relative to control muscles although basal levels were maintained [54]. In the heart, HB-EGF protein is required for normal cardiac function, inducing cardiomyocyte hypertrophy through an EGFR-ERK5-MEF2A-COX-2 pathway [56, 57] and has been implicated in the pathogenesis of cardiomyopathy [56, 58].

An emerging theme in our equine exercise transcriptomics and genomics research suggests a link between the exercise response and behavioural plasticity. For example, in the skeletal muscle transcriptome response to exercise training, neurological processes were the most significantly over-represented gene ontology (GO) terms, with the top three ranked GO terms being *Neurological system process* (*P* = 4.85 × 10^−27^), *Cognition* (*P =* 1.92 × 10^−22^) and *Sensory perception* (*P =* 4.21 × 10^−21^) [34]. Furthermore, in GWA studies we have demonstrated that genes (*HTR7*, *NTM* and *PCRP*) involved in behavioural plasticity are the most strongly associated with economically important traits in racing Thoroughbreds: precocity (early adaptation to racing) [45] and the likelihood of never racing [59]. For horses entering exercise training, behavioural plasticity enables the adaptation to an unnatural environment by reducing stress, with considerable variation in the abilities of horses raised in the same environment to adapt to stress. In rodents, ‘coping styles’ are under a high degree of genetic control [60]. However, it is becoming increasingly apparent that epigenetic regulatory mechanisms are key features of the modification of behavioural phenotypes and that there is likely a dynamic interplay between the fixed genome and the environment. In the brain, glucocorticoids are essential for adaptation to environmental stressors and are regulated by epigenetic modifications of glucocorticoid receptors that improve stress responses [61]. In response to exercise, glucocorticoids maintain energy homeostasis regulating the replenishment of glucose. The glucocorticoid receptor, which mediates the physiological and pharmacological actions of cortisol and other glucocorticoids, is the product of a single gene, *NR3C1*, which is also associated with obesity and metabolic syndrome. In the present study, *NR3C1* (14: 33819335–33923603) was within the flanking region of the second-ranked cluster on ECA14. The early post-natal environment is highly dependent on maternal input, with maternal care effects shown to have long-lasting influences on methylation status and the resulting behavioural phenotype [62]. Epigenetic modification of genes in the brain, including *NR3C1*, have been shown to be strongly associated with the response to early life stress [63].

As well as the prominent selected regions on ECA14, other genomic regions under selection peaks contained candidate genes that may contribute to the Australian racing phenotype. Flanking the selected region on ECA6 was *CLSTN3* (6: 34597086–34621970), which encodes calsyntenin-3, a synaptogenic adhesion molecule involved in neural development [64]. Calsyntenin-3 may play a role in control of locomotion since it has been shown to mediate neuro-adipose synaptic junction formation [65] and is required for GABAergic and glutamatergic synapse development [66]. The control of locomotion appears to be a key feature of selection for the Australian Thoroughbred phenotype. When we relaxed the criteria for inclusion of selected regions and defined selected regions among the top 1% of SNPs the third-ranked region (Table 2, S3 Table) on ECA16 (24.28–26.52 Mb) that also had the highest XP-EHH signal, centred on *ATXN7* (ataxin-7 gene, 16:25280990–25327556). This region has previously been identified as a region of interest (ROI 16: 24.16 Mb) in an investigation of selection signatures in racing Quarter horses [67]. A CAG repeat expansion in *ATXN7* causes spinocerebellar ataxia type 7 in humans, which has a significant tendency to be caused by paternal transmission of expanded alleles [68]. Spinocerebellar ataxia is a neurodegenerative inherited disease characterised, among other clinical signs, by poor coordination of muscle movement. In mice, *ATXN7* overexpression in the brain plays a role in the pathophysiology of attention deficit hyperactivity disorder (ADHD) [69, 70], a neurodevelopmental disorder characterized by varying levels of hyperactivity, inattention and impulsivity. In the Thoroughbred, the ataxin-7 protein may function in the coordination of gait; however, *ATXN7* is not significantly differentially expressed in skeletal muscle in the exercise or training response. Nonetheless, ataxin-7 function in the brain, in association with hyperactivity phenotypes, is intriguing to speculate considering that treatment with the ADHD drug amphetamine (AMPH) in an animal model for ADHD (SHR/NCrl) reduced hyperactivity but increased locomotor activity in control rats. *ATXN7* was one of only two differentially expressed genes (*ATXN7* and *PER2*) between the ADHD animal model and controls that were downregulated in response to AMPH treatment in SHR/NCrl. Therefore, we speculate that in the Thoroughbred, *ATXN7* may be involved in the control of locomotor activity.

Considering the variation in climatic conditions between Australia and other regions and the consequential effect on training regimes, it is interesting to note that based on Kyoto Encyclopedia of Genes and Genomes (KEGG) [71] and Gene Ontology (GO) annotations [72, 73] seven genes with functions in circadian rhythm—colony stimulating factor 2 (*CSF2*), epidermal growth factor receptor (*EGFR*), coagulation factor VII (*F7*), G protein subunit beta 3 (*GNB3*), histone deacetylase 3 (*HDAC3*), sirtuin 1 (*SIRT1*) and S-phase kinase associated protein 1 (*SKP1*)—were identified among the regions defined by the top 0.1% SNPs. This suggests there may be local adaptation to training at earlier hours of the day to avoid heat and the effect of unnatural lighting systems that are often used. An improved ability to cope with heat stress in Australia may be reflected by the presence of cell death inducing DFFA like effector A (*CIDEA*) [74–76] which is involved in metabolic rate, thermogenesis and lipolysis and sodium channel epithelial 1 alpha subunit (*SCNN1A*) [77–79] which is a component of sweat glands and has a function in the regulation of fluid balance, in the selected regions on ECA8 and ECA6 (S3 Table).

### GWAS for field-measured speed in young Thoroughbred horses

The requirement for achieving high speeds on the racetrack early in the two-year-old racing season in Australia is reflected in the racing calendar where the greatest value is placed on early two year old sprint races (≤ 1,200 m), with the principal races being the A$2 million Magic Millions 2YO Classic, A$3.5 million G1 Golden Slipper Stakes and A$1 million G1 Blue Diamond Stakes. Since there is a marked emphasis on selection for early two-year-old speed in Australia, next to further refine the results we compared the CSS results to a GWAS for field-measured speed in two-year-old horses in the early stages of exercise training. Previous studies have demonstrated improved power to detect complex trait loci by combining GWAS and selection signature mapping based on the same SNPs [40]. Here, early two-year-old speed was defined from a principal component analysis (PCA) of first sprint-training session (work day [WD], FWD) speeds obtained using GPS tracking equipment in a cohort of *n* = 179 (91 males, 88 females) horses-in-training in Ireland, a genetically, geographically and environmentally distinct cohort of horses to the samples used for the CSS analysis.

PC1 (FWD) and PC2 (FWD) explained 64.7% and 18.3% (total = 83%) of the variance in the six measured speed indices (V_peak_, Acc, aveSpr, Dist6a, Dist6b and Dist6; see methods) respectively (S3 Fig). Using 49,720 SNPs in a GWAS for PC1 (FWD), we observed a single peak on ECA14 centred around the top-ranked SNP BIEC2-255432 (g.35669710A>C; *P*_unadj_ = 3.22 × 10^−6^) (S5 Table, S4 Fig). Eight of the top 10 SNPs in the GWAS were located between 33.2–35.7 Mb, while the entire GWAS peak (13 SNPs) spanned a 4 Mb region (33.2–37.2 Mb), which overlapped with the top CSS peak. The top GWAS SNP ranked 44^th^ in the CSS analysis (ranked 9^th^ for *F*_*ST*_ test) and was ~700 kb from the top three CSS SNPs (S6 Table). Similar results were observed when relatedness between individuals was taken into account in the model. A single peak was identified with BIEC2-255432 (g.35669710A>C; *P*_unadj_ = 1.13 × 10^−5^) as the top-ranked SNP (S5 Fig).

As well as genes contained within the CSS peaks, the GWAS peak encompassed a large protocadherin gene cluster. Most protocadherin genes are clustered together at a small number of genomic loci [80]. The protocadherin gamma genes are expressed principally in neural tissue and may provide guidance for axon binding [33, 80]. Differential expression of these genes in individual neurons ensures cellular diversity in neural circuit formation [81]; for instance, protocadherin-alpha and protocadherin-beta are known to function cooperatively for neuronal survival [82]. It has been suggested that their expression at the muscle side of the neuronal synapse may facilitate axon guidance towards muscle to facilitate reinnervation at the neuromuscular junction [83]. It has been shown that the γ2 subunit of the GABA-A receptor directly interacts with the product of *PCDHGC5* in the rat brain [84] and it has been suggested that *PCDHGC5* plays a role in GABAergic synapse formation or GABA-A receptor clustering. In humans SNPs close to *PCDHB15* and *PCDHGA1* have been associated with carotid artery intima media thickness progression, which is diagnostic for the presence of atherosclerosis [85]. *PCDH12*^-/-^ mice have altered structural and functional modifications to the arteries and age-dependent vascular phenotype variation has been observed for the carotid artery. In humans the corresponding gene cluster containing *PDHA12*, *PCDHAC2*, *PCDHB5*, *PCDHB6*, *PCDHB12*, *PCDHGA6*, *PCDHGB7*, *PCDHGA11* and *PCDH12* has been implicated in idiopathic pulmonary arterial hypertension [86]. In the Thoroughbred, we have previously observed differential expression of four (*PCDH12*, *PCDH17*, *PCDH19* and *PCDHB15*) of the 80 protocadherin genes in the skeletal muscle response to exercise [34], two of which (*PCDH12* [protocadherin 12, 1.3-fold, *P* = 3.60 × 10^−5^] and *PCDHB15* [protocadherin beta 15, 1.4-fold, *P* < 0.05]) were located within the GWAS peak.

Interestingly, in an investigation focusing on the *NRC31* gene region for variation in maternal care style in the rat, the highest differential methylation response was observed for the orthologous chromosomal region containing the protocadherin gene cluster [87]. It has been suggested that epigenetic responses to maternal care are coordinated not at a single gene locus but rather across broad genomic regions [87]. It is therefore intriguing to speculate that epigenetic modification of genes across the large selected region on ECA14 is modulated by the early care environment of the Thoroughbred, which may influence the stress response and impact on early adaptation to the racing and training environment. In Australia, where the emphasis is on early two-year-old racing, the ability of a young horse to adapt to the rigours and stresses of the environment may be of greater importance than in other racing regions. The previous observations of epigenetic modifications in the ECA14 region suggests that the behavioural phenotype of young horses may be contributing to selection.

### Association of ECA14 SNPs with elite racing performance in Australian Thoroughbreds

To establish whether selection acting at the ECA14 locus in Australian Thoroughbreds contributes to variation in racecourse performance we performed association tests for a set of 109 higher density SNPs in the ECA14 region (35000778–35999735) ascertained from the Affymetrix Axiom 670k genotyping array. Allele frequencies among elite Australian horses (*n* = 109, CPI > 2, *i*.*e*. earned more than double the average) were compared to low performing Australian horses (*n* = 232, CPI < 0.56, *i*.*e*. earned less than half the average), and similarly elite European horses (*n* = 242) were compared to low performing European horses (*n* = 339). Following correction for multiple testing two SNPs associated with the elite performance phenotype in Australian horses were identified (14: 35578513, *P* = 0.0024; 14: 35758560, *P* = 0.0103) (S7 Table, S8 Table). The SNP-35578513 was located within the *PCDHGC5* (protocadherin gamma subfamily C, 5) gene. The frequency of the favourable G-allele at SNP-35578513 was 0.73 in elite and 0.54 in non-elite Australian horses and was also observed at a higher frequency in elite (0.78) compared to non-elite (0.72) European horses. The G-allele frequency in Australia was lower (0.59, *n* = 341) compared to Europe (0.77, *n* = 581) indicating that the unfavourable allele may be inadvertently proliferating in Australia due to its presence in prominent sire lines.

## Conclusion

We have successfully applied the CSS approach to identify genomic regions subject to selection in Australian Thoroughbreds and identified underlying candidate genes that have been captured by breeders as a consequence of artificial selection over generations to maximise success in the Australian racing ecosystem. By combining our results with a GWAS for a measured exercise phenotype and cross-referencing with previously reported transcriptomics data, we have identified a genomic region on ECA14 that is a highly plausible candidate for the effects of local adaptation in the Thoroughbred. Our results point to selection for genes involved in the control of synapse formation at the neuromuscular junction that may be important for locomotion and genes that may contribute to behavioural plasticity. However, while individual gene-specific variants appear to be segregating with performance, it is likely that a suite of functionally related genes contribute to the population-wide variation in the racing phenotype that is adapted to the specific racing requirements in Australia. Our results illustrate the genomic plasticity among populations that are under human-mediated selection.

Here, we have observed that the genomic locus subject to the strongest selection in the Australian population was also associated with early two-year old speed in an entirely independent cohort of horses. Furthermore, the association of the g.35578513 SNP with elite racing performance in a large cohort of horses points specifically to a contribution to the racing phenotype from allelic variation at this locus. While further functional experiments are warranted to understand the underlying physiological endophenotypes contributing to the Australian racing phenotype, these results have the potential to be used for marker-assisted selection to screen for horses best suited for the Australian racing ecosystem.

## Methods

### Samples

Blood samples were obtained from *n* = 99 Thoroughbred horses that were born in Australian (*n* = 49) and in other regions including Europe, North America and South Africa (*n* = 50) for isolation of DNA for the purposes of genetic testing for the *MSTN* g.66493737 SNP. Consent was given for use of the samples in research. Samples were anonymised.

### Composite Selection Signals (CSS) cohorts

The Australian (*n* = 49) versus non-Australian (*n* = 50) comparator cohorts comprised elite horses and had *MSTN* g.66493737 T/C genotypes proportionate to the distribution among the local regional population [1]. Elite was defined as having a Comparative Performance Index (CPI) >5 (which equates to earnings ~€200,000 - €300,000). Both groups had similar mean racing performance metrics based on the CPI (Table 3).

**Table 3:** Performance metrics and *MSTN* g.66493737 SNP genotypes among the comparator cohorts. All horses were elite performers. Within each cohort there was a similar *MSTN* genotype distribution to that observed previously within the regional population. CPI–Comparative Performance Index.

**Cohort**	**Comparative Performance Index (CPI)**			***MSTN* genotype (n)**		
	**mean**	**min**	**max**	**CC**	**CT**	**TT**
Aus	26.92	5.01	215.15	23	15	11
non-Aus	19.9	5.04	173.37	15	32	3

### Genotyping & QC

Genomic DNA was extracted from whole blood using the Maxwell 16 automated DNA purification system (Promega, Madison, WI). Horses were genotyped using two high-density SNP genotyping arrays: Illumina Equine SNP70 BeadChip (Illumina, San Diego, CA) and Axiom Equine Genotyping Array (Axiom MNEC670) (Affymetrix, Santa Clara, CA). Concordant SNPs derived from the SNP70 and SNP670 arrays were used for the analysis. Individuals and SNPs were subject to a genotyping threshold of 95%. SNPs that failed quality-control were imputed using BEAGLE [version: 3.3.2] [88]. A genetic sex check and minor allele frequency threshold of > 0.01 were also included as quality-control. Previously ten horses were genotyped on both the SNP70 and SNP670 array and post imputation concordance was found to be > 99% [89]. After quality-control, 46,478 SNPs were derived for CSS analyses and for association testing with physiological phenotypes. For a set of *n* = 922 horses genotyped on the Axiom MNEC670 array, 109 SNPs in the region EqCab2 14:35000000–14:36000000 were extracted to test for associations with racetrack performance.

### Composite selection signal (CSS) method

Population stratification among the comparison cohorts was examined by performing Principal Component Analysis (PCA) using smartPCA from the EIGENSOFT package (version 4.2) [90]. Group differences were calculated by using the command (—ibs-test) in PLINK[91], with respect to a binary phenotype (Aus TB v NonAus TB) based upon pairwise identity-by-state (IBS) distance between all individuals. To validate the CSS approach, a dataset was generated using best race distance as phenotype for European elite horses to identify the *MSTN* region when comparing short ≤8f (i.e. 5-8f, 1600m) to long distance >8f (i.e. 9+ f, 1800m) horses. A SNP (ECA18: 66493737) tagging the *MSTN* gene located on ECA18 is strongly associated with optimum race distance in TB [92]. Best Race Distance (BRD) was defined as the distance of the highest value race won by the horse or if a non-winner the highest value race in which a horse was placed. Unplaced horses were not included. The phenotype BRD-Elite includes only Elite winners and is a more accurate phenotype with higher heritability [1]. The CSS comparison for the European elite horses included *n* = 50 elite performers in short distance races versus *n* = 50 elite performers in long distance and an equal number of males and females was included in each comparator group.

The CSS approach was developed to investigate genomic signatures of selection and has been successful at localizing genes for monogenic and polygenic traits under selection in livestock [6, 8, 93]. The CSS uses fractional ranks of constituent tests and does not incorporate the statistics with *P* values, allowing a combination of the evidence of historical selection from different selection tests. For the present study, the CSS combined the fixation index (*F*_ST_), the change in selected allele frequency (Δ*SAF*) and the cross-population extended haplotype homozygosity (*XP-EHH*) tests into one composite statistic for each SNP. *F*_ST_ statistics were computed as the differentiation index between the population(s) of interest (*i*.*e*. selected) and the contrasting/reference population(s) (*i*.*e*. non-selected). *XP-EHH* and Δ*SAF* statistics were computed for the selected population(s) against the reference population. The CSS were computed as follows:

1. For each constituent method, test statistics were ranked (1, …, n) genome-wide on n SNPs.
2. Ranks were converted to fractional ranks (r´) (between 0 and 1) by 1/ (n + 1) through n / (n + 1).
3. Fractional ranks were converted to z-values as z = Φ-1(r´) where Φ-1(⋅) is the inverse normal cumulative distribution function (CDF).
4. Mean z scores were calculated by averaging z-values across all constituent tests at each SNP position and *P*-values were directly obtained from the distribution of means from a normal N (0, m^–1^) distribution where m is the number of constituent test statistics.
5. Logarithmic (–log_10_ of *P*-values) of the mean z-values were declared as CSS and were plotted against the genomic positions to identify the significant selection signals.
6. To reduce spurious signals, the individual test statistics were averaged (smoothed) over SNPs across chromosomes within 1 Mb sliding windows.

According to the approach proposed by [6], significant genomic regions were defined as those that harbour at least one significant SNP (top 0.1%) surrounded by at least five SNPs among the top 1%. Here, we relaxed the stringency to define significance as regions harbouring at least five SNPs among the top 1% since the numbers of regions would otherwise be small (i.e. ~48 SNPs). Also, since linkage disequilibrium extends up to 0.4 Mb [94] in the Thoroughbred, we considered 1 Mb sliding windows reasonable in this population. Therefore, SNPs among the top 1% smoothed CSS values within the sliding windows were considered significant.

### Identification of selected genomic regions and candidate gene mining

To localise genomic regions and genes under selection, we defined significant selected regions as those that consisted of at least five SNP among the top 0.1% (*i*.*e*. 48 SNPs). Consecutive clusters spaced < 1 Mb apart were merged into a single cluster. Genes underlying the selection peaks as well as flanking regions (± 0.5 Mb) were mapping to an annotated protein coding gene list from EquCab2.0 downloaded from Ensembl (accessed: 2018-10-23). These genes were then examined for evidence of functional significance. Considering the LD in the Thoroughbred and the observed extended haplotypes in regions known to be influenced by strong selection [3] we also identified genes among the top 1% of SNPs (*i*.*e*. 480 SNPs). The variants identified in the main region of interest on ECA14 were mapped to EquCab 3 positions to confirm correct annotation of the protocadherin gene cluster.

### Exercise physiology phenotyping

**Exercise tests:** WD were performed on a woodchip, 1,500 m, uphill, all-weather gallop track, with the final 800 m straight on a 2.7% incline [95]. Prior to each WD, horses were walked on an automated horse walker for 30–60 min, followed by 5–10 min of walking in hand. Warm-up under saddle consisted of a 300 m walk followed by a 700 m trot and slow canter down the incline of the track. A short period of walk followed. The sprint portion of the WD consisted of the horses galloping at high-intensity for 800−1,000 m.

**Experimental measurements:** Velocity (V) and distance were measured using a STATSports Viper GPS monitoring system (STATSports Technologies Ltd. Newry, Northern Ireland). Speed indices originally described by [95] were derived from the GPS measurements taken during the sprint portion of the WD, defined as when the horse first exceeded 5 m/s until reaching peak velocity (V_peak_). Correlations among speed indices were determined using Pearson’s correlation. PCA was performed using peak velocity (V_peak_), acceleration time (Acc), average sprint velocity (aveSpr), distance covered in the 6 s proceeding V_peak_ (Dist6a), distance covered in the 6 s preceding V_peak_ (Dist6b) and distance covered in the 6 s preceding and proceeding V_peak_ (Dist6) as input variables (Table 4), using ‘*princomp*’ within the R environment [version: 3.4.1] [96].

**Table 4:** Definitions of speed indices derived from GPS measurements used for the development of principal components.

Speed Index	Definition
V_peak_	Peak velocity (m/s)
Acc	Time taken (s) from when the horse first exceeded 5m/s in the sprint period until V_peak_ was reached
aveSpr	Average velocity (m/s) during the sprint period
Dist6a	Distance (m) covered in the six seconds post- V_peak_
Dist6b	Distance (m) covered in the six seconds preceding V_peak_
Dist6	Distance (m) covered in the six seconds before and after reaching V_peak_

PC1 was used as the phenotype. All horses were < 3yo and had not completed > 4 WDs prior to measurement. The value of PC1 from the earliest recording (*i*.*e*. first WD, FWD) was used (PC1(FWD)).

**GWAS:** Tests of genome-wide association were performed for the quantitative phenotype PC1 (FWD) (*n* = 179) in PLINK with sex as a covariate [91]. Results were visualised in R using the package qqman [97]. Mixed model analyses (polygenic and mmscore [98] were also carried out to account for the relatedness of individuals. The threshold for genome-wide significance was determined using the Bonferroni correction based on the effective number of independent loci (*M*_e_) using the Genetic Error Calculator [version 0.2] [99], with the threshold for genome-wide significance set at $\frac{0.05}{M_{e}}$ and the suggestive threshold for association $\frac{1}{M_{e}}$. The effective number of loci was *M*_e_ = 20,661, which gave a suggestive significance threshold (*P* = 4.8 x 10^−5^) and a genome-wide level for significance of association (*P* = 2.4 x 10^−6^).

### Racing performance analysis

To provide denser coverage across the ECA14 region, *n* = 922 Thoroughbred horses were genotyped on the Affymetrix 670 genotyping array. The following phenotypes were used: 1) High performing Australian (*n* = 109, CPI > 2, *i*.*e*. earned more than double the average) compared to low performing Australian horses (*n* = 232, CPI < 0.56, *i*.*e*. earned less than half the average); 2) High performing European (*n* = 242, CPI > 2, *i*.*e*. they earned more than double the average) compared to low performing European horses (*n* = 339, CPI < 0.56, *i*.*e*. they earned less than half the average). Within all cohorts only *MSTN* C/C and C/T horses were included. Tests of genetic association with the elite (high performing) phenotype were performed for both Australian and European sets of horses in PLINK for *n* = 109 SNPs within a 1 Mb region (35–36 Mb) on ECA14. The following QC thresholds were applied to each of the association tests: minor allele frequency > 0.05 and individual call rate > 95%.

### Ethics statement

University College Dublin Animal Research Ethics Committee approval (AREC-P-12-55-Hill) and a licence from the Department of Health (B100/3525) for samples and data collected for the horses-in-training cohort was obtained and informed owner consent for use of samples in research was obtained for all horses.

## Supporting information

S1 Fig Principal component analysis (PCA) for Australian versus non-Australian cohort. (PDF)

S2 Fig Validation of Composite selection signal (CSS) approach using best racing distance (BRD) as phenotype comparison. The second strongest signal mapped to ECA18 and contained *MSTN*.
(PDF)

S3 Fig Schematic representation of speed indices derived from GPS monitoring during work days (two-year-olds, ≤4 WDs, single measurement). a) Measured speed indices include (V_peak_, Acc, aveSpr, Dist6a, Dist6b and Dist6). b) Phenotypes were summarised using principal component analysis and PC1 and PC2 explained 83% of the variance among speed indices. PC1 defined the ‘early 2yo speed’ phenotype for the GWAS.
(PDF)

S4 Fig Manhattan plot for tests of genome-wide association (GWA) for PC1. The ECA14 region was identified as a candidate locus for early two-year-old speed. The top SNP in the GWAS (BIEC2-255432) was located at 14:g.35669710.
(PDF)

S5 Fig Manhattan plot for tests of genome-wide association (GWA) for PC1 following correction for relatedness which identified the ECA14 region as a candidate locus for early two year old speed. The top SNP in the GWAS (BIEC2-255432) was located at 14:g.35669710.
(PDF)

S1 Table Validation of Composite selection signature (CSS) approach using Best race distance (BRD) as phenotype comparison. The second strongest signal mapped to ECA18 and contained *MSTN*.
(XLSX)

S2 Table Genomic regions under selection in Australian horse populations. Clusters of a minimum of five top 0.1% SNPs within a window spanning 1 Mb genomic regions were defined as regions under selection.
(XLSX)

S3 Table Genomic regions under selection in Australian horse populations. Clusters of a minimum of five top 1% SNPs within a window spanning 1 Mb genomic regions were defined as regions under selection.
(XLSX)

S4 Table Examination of expression QTL (eQTL) at the ECA14 region in skeletal muscle identified *ARHGAP26* (Rho GTPase activating protein 26, GRAF-1, 14: 33978983–34396100) to be a strong candidate on which selection is acting. Thirty-four significant (*P*_FDR_ < 0.05) *cis*- and *trans*-eQTL were identified in the ECA14 region in resting and post-exercise equine skeletal muscle samples.
(XLSX)

S5 Table Using 49,720 SNPs in a GWAS for PC1 (FWD) a single peak on ECA14 centred around the top-ranked SNP BIEC2-255432 (g.35669710A>C; *P*_unadj_ = 3.22 × 10^−6^; *P*_GC_ = 1.10 × 10^−5^). (XLSX)

S6 Table Genes located in the top ECA14 GWA region which overlapped with the CSS peak. The region spans from 33.2–35.6Mb.
(XLSX)

S7 Table Association tests for a set of 109 SNPs in the ECA14 region (35000778–35999735) ascertained from the Affymetrix Axiom 670k genotyping array in elite Australian and non-elite Australian horse population. Allele frequencies among elite Australian horses (*n* = 109, CPI > 2, *i*.*e*. earned more than double the average) were compared to low performing Australian horses (*n* = 232, CPI < 0.56, *i*.*e*. earned less than half the average). Following correction for multiple testing a SNP associated with the elite performance phenotype in Australian horses was identified (EqCab 14: 35578513, *P* = 0.00238).
(XLSX)

S8 Table Association tests for a set of 109 SNPs in the ECA14 region (35000778–35999735) ascertained from the Affymetrix Axiom 670k genotyping array in elite European and non-elite European horse population. Allele frequencies among elite European horses (*n* **=** 242, CPI > 2, *i*.*e*. earned more than double the average) were compared to low performing European horses (*n* **=** 339, CPI < 0.56, *i*.*e*. earned less than half the average). Following correction for multiple testing no SNPs associated with the elite performance phenotype in European horses was identified.
(XLSX)
