# Supplementary figures and images for: Selection in Australian Thoroughbred horses acts on a locus associated with early two-year old speed
Source: PLoS One. 2020 Feb 12;15(2):e0227212. doi: 10.1371/journal.pone.0227212 (PMC7015314; doi:10.1371/journal.pone.0227212)

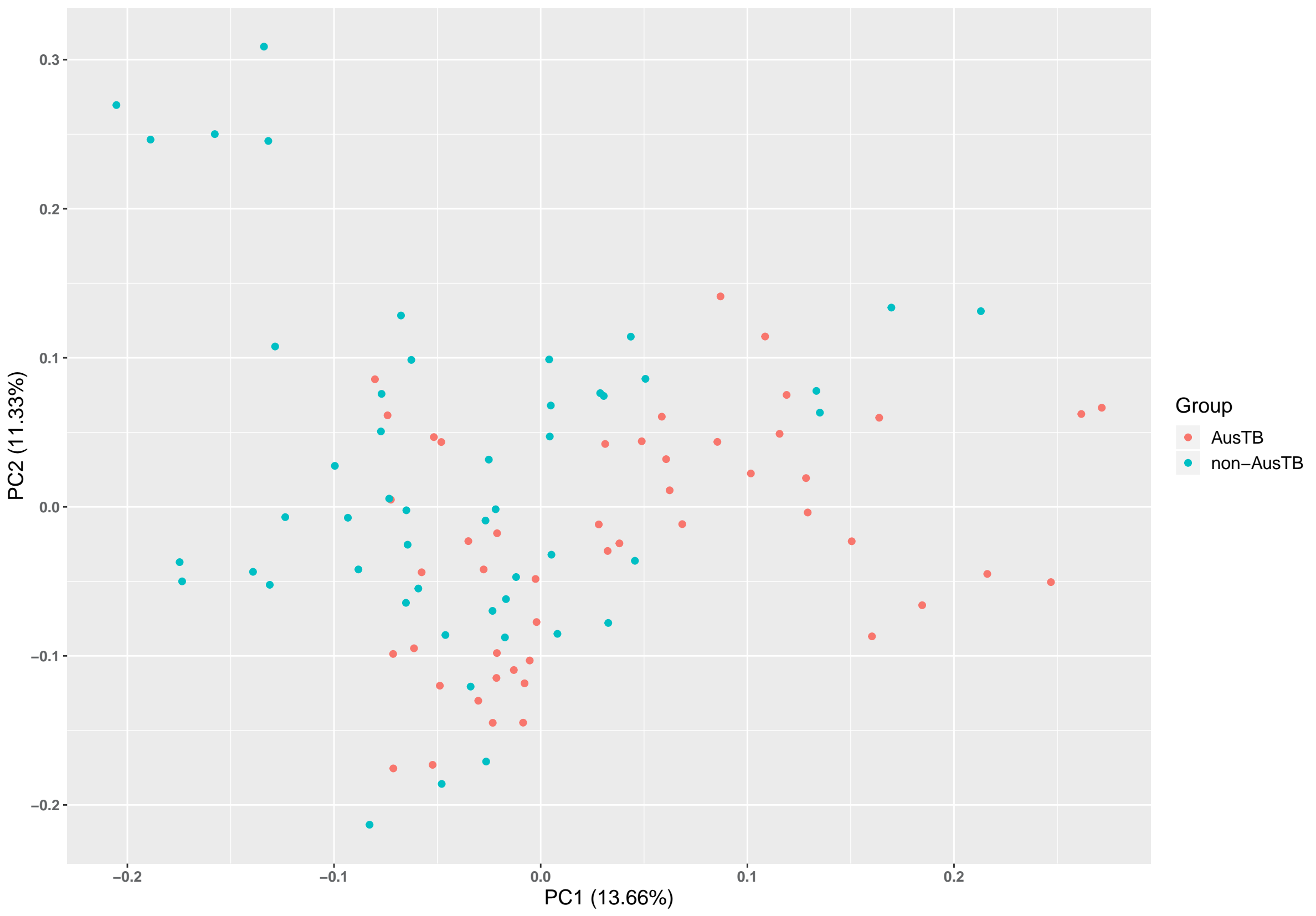

Supplement: S1 Fig — (PDF) [file pone.0227212.s001.pdf]

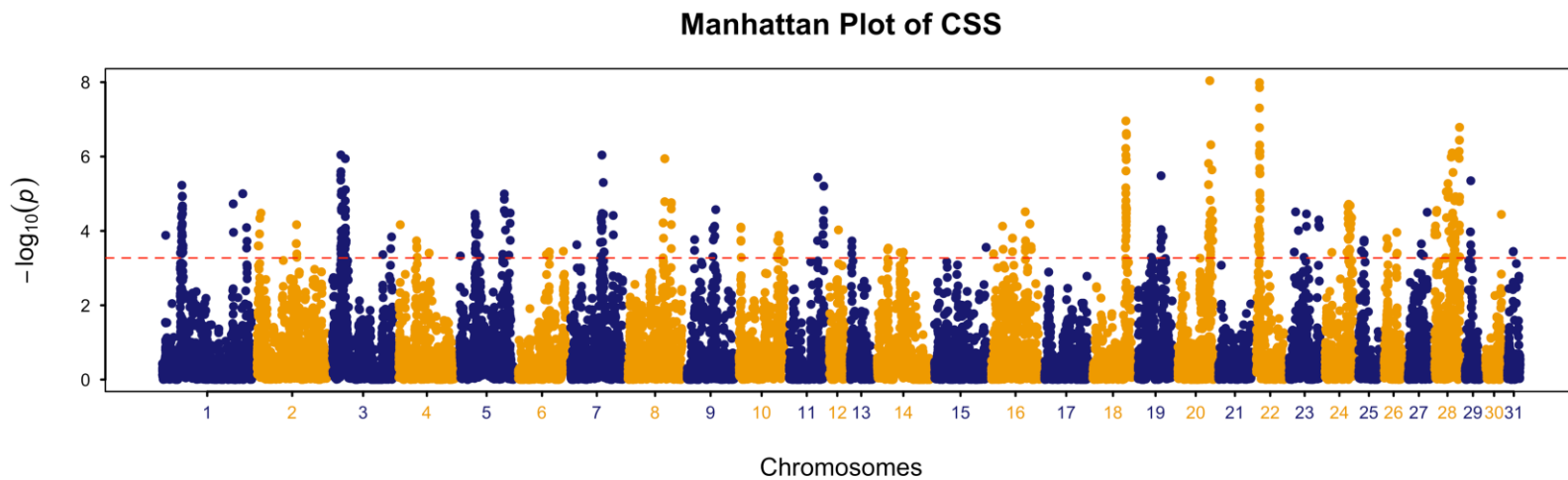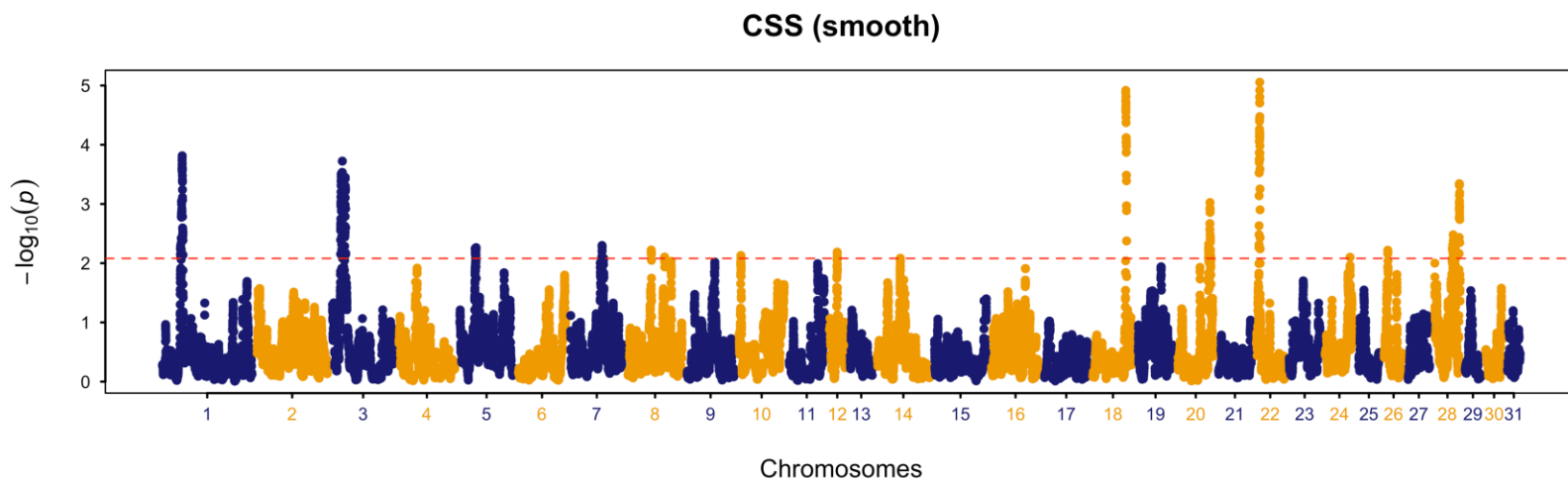

Supplement: S2 Fig — The second strongest signal mapped to ECA18 and contained MSTN. (PDF) [file pone.0227212.s002.pdf]

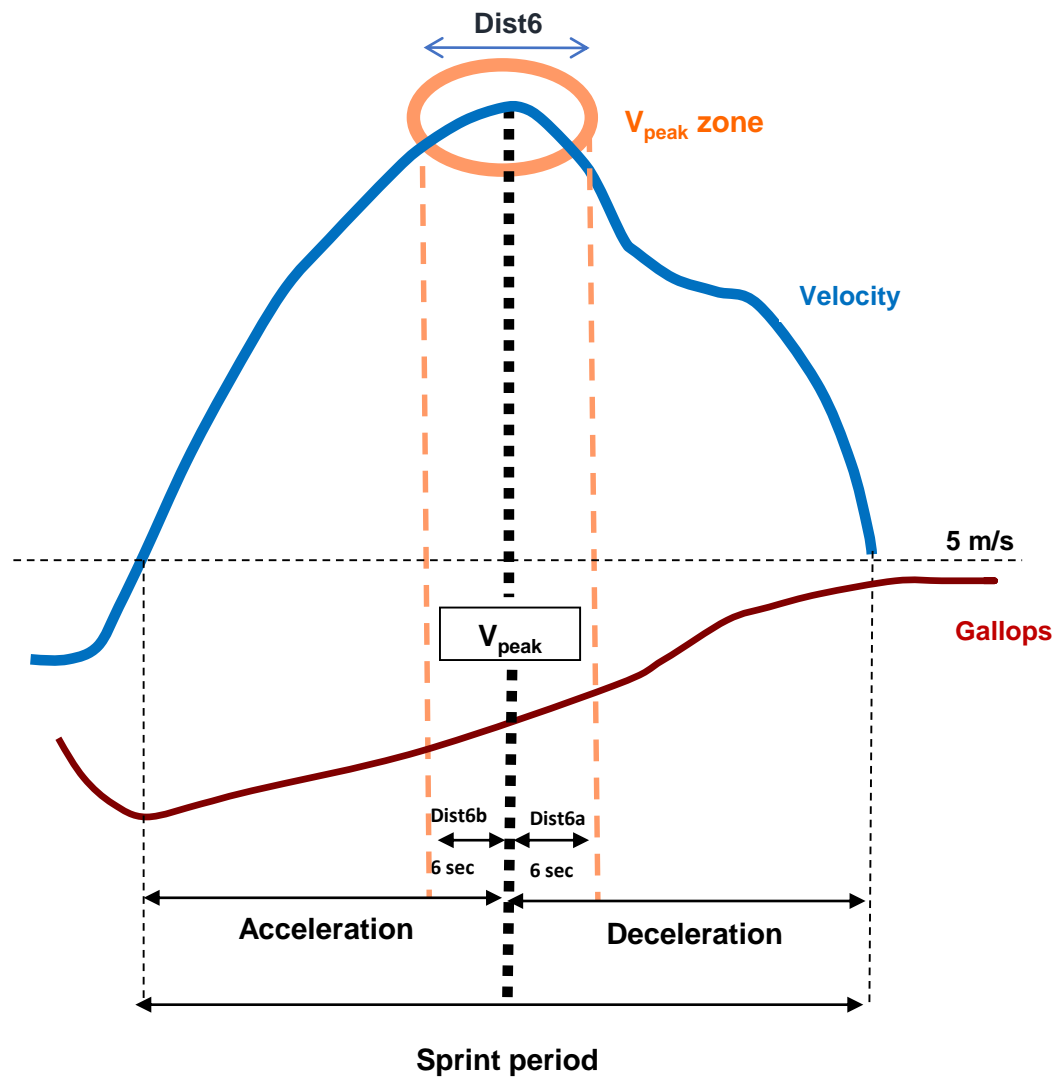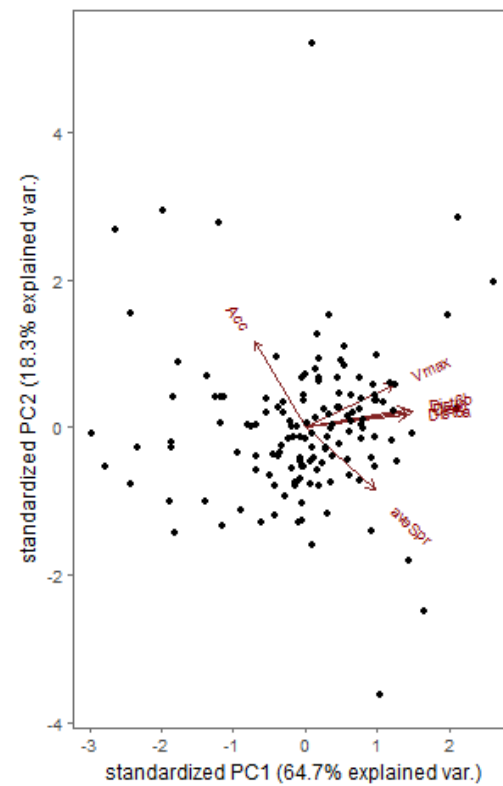

Supplement: S3 Fig — a) Measured speed indices include (Vpeak, Acc, aveSpr, Dist6a, Dist6b and Dist6). b) Phenotypes were summarised using principal component analysis and PC1 and PC2 explained 83% of the variance among speed indices. PC1 defined the ‘early 2yo speed’ phenotype for the GWAS. (PDF) [file pone.0227212.s003.pdf]

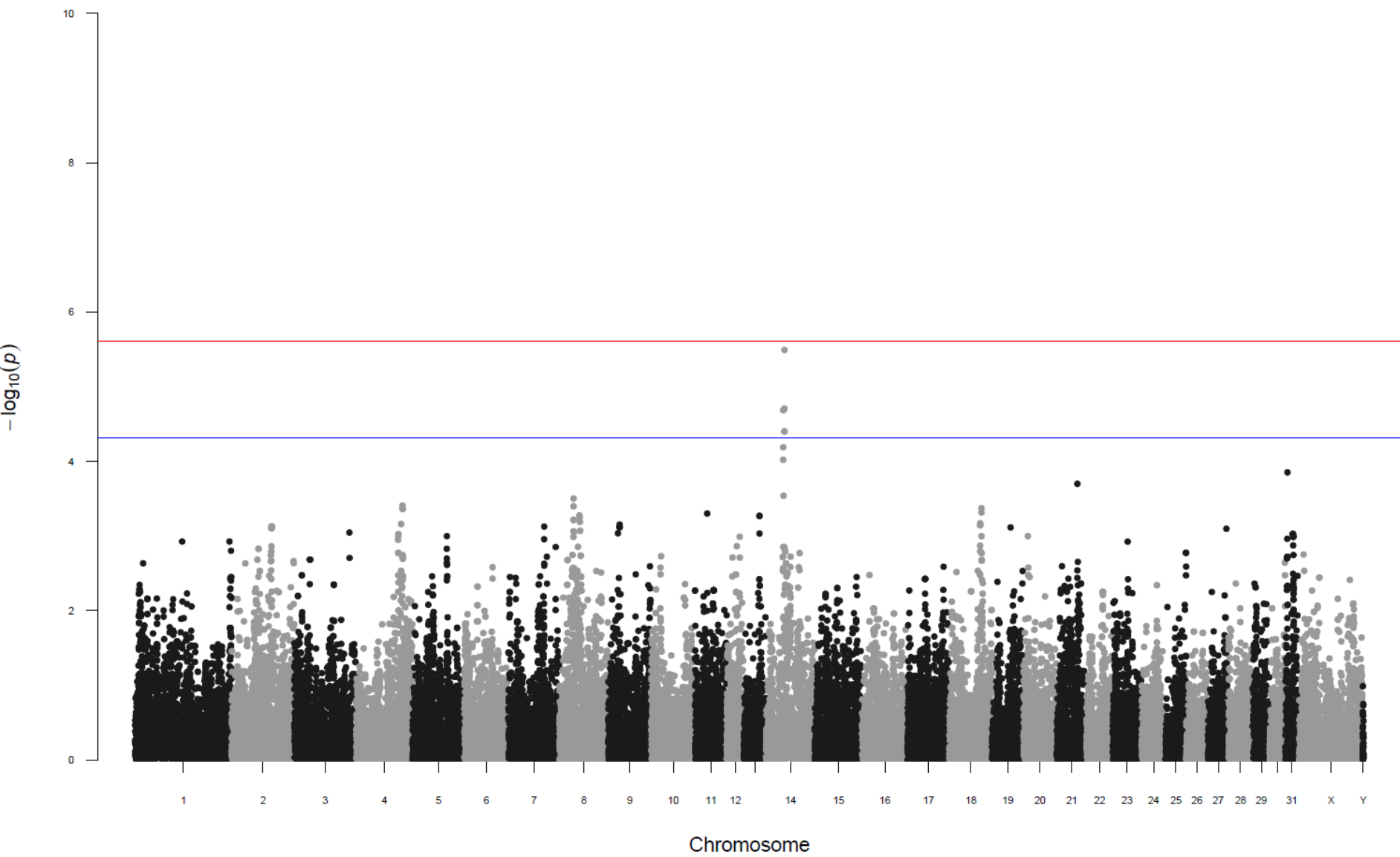

Supplement: S4 Fig — The ECA14 region was identified as a candidate locus for early two-year-old speed. The top SNP in the GWAS (BIEC2-255432) was located at 14:g.35669710. (PDF) [file pone.0227212.s004.pdf]

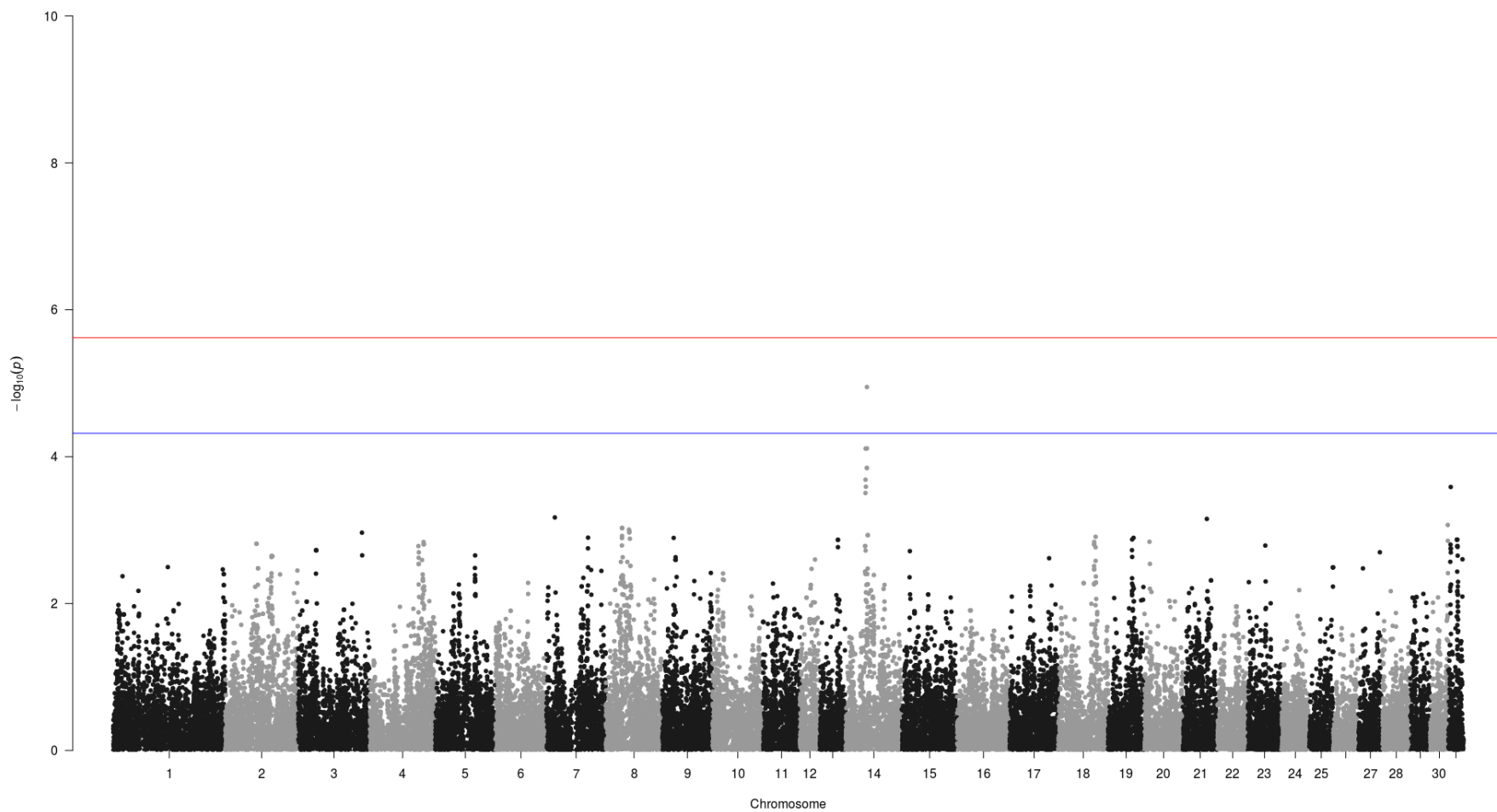

Supplement: S5 Fig — The top SNP in the GWAS (BIEC2-255432) was located at 14:g.35669710. (PDF) [file pone.0227212.s005.pdf]
